# Synthesis and Characterization of Some Chiral Metal-Salen Complexes Bearing a Ferrocenophane Substituent

**DOI:** 10.3390/molecules14114312

**Published:** 2009-10-26

**Authors:** Angela Patti, Sonia Pedotti, Francesco Paolo Ballistreri, Giuseppe Trusso Sfrazzetto

**Affiliations:** 1 Istituto di Chimica Biomolecolare del CNR- Via Paolo Gaifami 18, I-95126 Catania, Italy; 2 Dipartimento di Scienze Chimiche, Università di Catania, Viale Andrea Doria 6, I-95125 Catania, Italy

**Keywords:** chiral salen ligands, [5]ferrocenophane, metal complexes, circular dichroism spectra

## Abstract

The *C*_2_-symmetrical “salen” ligand (+)-**9** bearing two [5]ferrocenophane substituents has been prepared in five steps starting from readily available diacetylferrocene, *p*-hydroxybenzaldehyde and (*R*,*R*)-*N*,*N’*-diphenylethylenediamine. Reaction of (+)-**9** with Mn(OAc)_3_, Co(OAc)_2_ ZnEt_2_ or UO_2_(OAc)_2_ gave the corresponding metal-complexes which were characterised by spectroscopic methods.

## 1. Introduction

Chiral *N*,*N’*-bis(salicylidene)ethylendiamine (salen) compounds are very popular ligands because of their easy formation and rich coordination chemistry with a large variety of metal ions, that has allowed their use as catalysts in different asymmetric reactions [1,2]. The Mn(salen) complexes have been mainly employed in the enantioselective epoxidation of *cis*-olefins [3,4] and their efficiency in the asymmetric induction has been related with their structural features [5,6]. The asymmetric ring opening of epoxides and the hydrolytic kinetic resolution of epoxides are actively promoted by Cr- or Co(salen) complexes [7,8,9], and the latter have been recently found interesting application in the stereoselective recognition of aminoalcohols and aminoacids [10,11].

Asymmetric alkylation of α-aminoacid enolates can be carried out in the presence of Cu(II)- or Co(II)salen catalysts [12], both possessing square-planar coordination, whereas the alkynylation of ketones has been performed with moderate enantioselectivity using Zn(salen) complexes [13,14].

More recently the salen framework has been used as building block in the creation of sophisticated homogeneous, multimetal catalysts and some salen complexes have been specifically designed for their application as functional materials [15]. The incorporation of “salen” moieties into macrocyclic structures able to give rise to supramolecular interactions [16] and the synthesis of salen compounds bearing Lewis acid or Lewis base activating groups are currently investigated for the development of more active catalysts [17,18]. 

Ferrocenyl compounds display unique electronic, steric and chemical properties which have been exploited in the synthesis of a great variety of derivatives and their useful application in different field of chemistry is well documented [19,20]. Our interest in the preparation of chiral ferrocenes [21,22] as well as in the development of new salen catalysts [23,24] prompted us to plan the synthesis of novel compounds bearing both these structural elements. 

Some achiral ferrocene-containing salen ligands have been reported and their coordination behaviour [25,26] or catalytic activity [27] investigated. Enantiopure derivatives, potentially useful in asymmetric homogeneous catalysis, have been prepared by Bildstein and coworkers [28] incorporating planar chiral ferrocenes into the salen backbone. 

The synthesis of two salen-type ligands having the ferrocenyl moiety on the chiral diimine bridge have been also described and one of them showed an unusual selectivity in the epoxidation of *cis*-olefin affording *trans*-epoxide as main isomer, but the asymmetric induction was not satisfactory [29]. Here we describe the synthesis of a chiral C*_2_*-symmetrical ligand, bearing a ferrocene system as substituent on the 5,5’-positions of the salicylidene unit, together with the spectroscopic characterization of its complexes with some metals. 

## 2. Results and Discussion

### 2.1. Synthesis of the ligand and its metal complexes

Since the steric and electronic features of the 3,3’- or 5,5’-substituents of a salen catalyst can exert some influence on its efficiency in the asymmetric induction process [5,6,30,31], the introduction of a ferrocenyl substituent, with its peculiar properties, on the salicylaldeyde moiety was considered in the design of novel salen ligands. Among the possible ferrocenyl substituents, the ferrocenophane system, in which the two cyclopentadienyl rings are joined by an alkyl chain, appears attractive due to its rigidity and the presence of a cavity that could play a role in determining the direction of approach of the reagents to the catalytic site. 

**Scheme 1:**
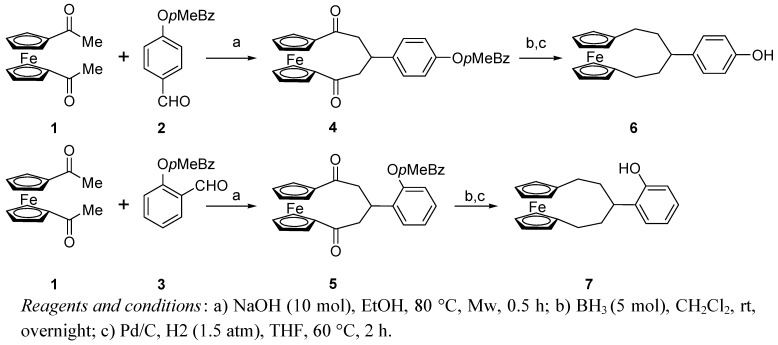
Synthesis of [5]ferrocenophane compounds.

The synthesis of 1,5-dioxo-3-substituted [5]ferrocenophanes has been recently reported to occur in high yield by simple mixing of 1,1’-diacetylferrocene **1** and a suitable aldehyde in NaOH/ethanol solution under microwave irradiation [32]. The reaction can be also applied to salicylaldehydes, but better results were obtained using the corresponding *O*-benzylderivatives, so 2-hydroxybenzaldehyde or 4-hydroxybenzaldeyde were reacted with *p*-methylbenzylbromide in acetone in the presence of CsCO_3_ at room temperature for 12 h to afford compounds **2** and **3** in almost quantitative yields. The treatment of **1** with **2** in the reported conditions gave the [5]ferrocenophane **4** as a yellow solid, practically insoluble in all the common organic solvents, even after its *O*-deprotection. Deoxygenation of **4** by treatment with excess of borane in CH_2_Cl_2_ and removal of the benzyl group with hydrogen over Pd/C gave the alkylderivative **6** in 85% overall yield, this compound being soluble and more suitable for the following step of formylation on the aryl ring. The same reaction sequence was applied to prepare the isomeric [5]ferrocenophane **7 **starting from **1 **and **3 **(Scheme 1). 

Firstly a Gross-Rieche formylation was attempted on derivative **6**, but it was quickly converted into a complex mixture of products and no selectivity for the formation of monoformyl derivative **8** was achieved either varying the reagent ratios or the reaction temperature. In the Riemar-Tiemann conditions, a selective 25% conversion of **6** into **8** was evidenced after 24 h, so the reaction was quenched at this stage and purified to recover the unreacted substrate that could be recycled and after four reaction cycles, the target aldehyde **8** was obtained in 55% overall yield. 

**Scheme 2:**
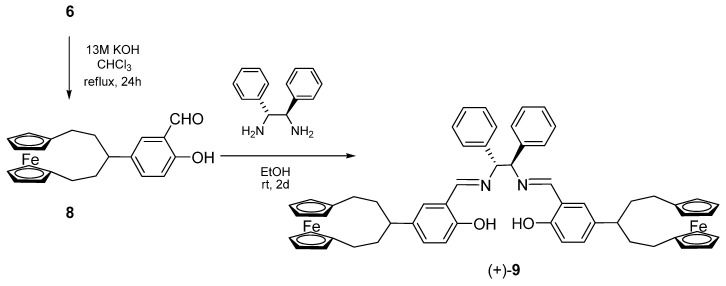
Synthesis of the salen ligand bearing ferrocenophane substituents.

Unfortunately, all the attempts at *ortho*-formylation of **7** using different procedures failed and the *para*-substitution product was formed in CHCl_3_/KOH system, so that we were only able to prepare ligand (+)-**9** by condensation of **8** with (+)-(1*R*,2*R*)-diphenylethylendiamine [(*R*,*R*)-dpen] (Scheme 2).

The symmetrical structure of (+)-**9** was confirmed by its ^1^H-NMR spectrum and diagnostic resonances at δ 8.41 and 13.16 for the iminic and phenolic protons, respectively, together with a singlet at δ 4.76 for the protons on the diimine bridge were observed. The methylenic protons on the ferrocenophane bridge gave rise to four separate multiplets in the range 1.8-2.4 ppm whereas the methinic signal was overlapped by the cycplopentadienyl resonances. Complete assignment of the carbon resonances by 2D-NMR spectra and molecular peaks at *m/z* 924.2 in the ESI-MS spectrum also supported the structure of ligand (+)-**9**.

The treatment of (+)-**9** with Mn(OAc)_3_ or Co(OAc)_2_ in EtOH gave the corresponding complexes (−)-**10** and (+)-**11** in high yield; due to their paramagnetic nature they were characterised by their ESI-MS and circular dichroism spectra (*vide infra*). 

**Figure 1:**
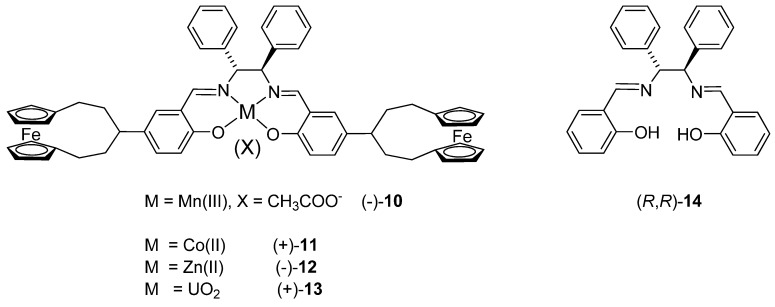
Metal complexes of salen ligand (+)-**9**.

The structure of salen-Zn complex (–)-**12**, prepared by reacting the free ligand (+)-**9** with Et_2_Zn in toluene, was mainly established by NMR analysis. The ^1^H-NMR spectrum of (–)-**12** in CDCl_3_ was fully unresolved whereas in (CD_3_)_2_SO all the resonances could be assigned and the symmetric nature of the complex was evidenced; the main features of the spectrum are the absence of the phenolic signal and a slight upfield shift for both the resonances of azomethine and *N*-methinic protons compared with those of the free ligand. Complexes **10**-**12** (Figure 1) are quite stable and have the potential to be used as catalysts in asymmetric synthesis.

Salen-uranyl(VI) complexes have been prepared as models for the pseudo-octahedral salen Mn=O species responsible for the selective oxygen transfer in the asymmetric epoxidation of olefins [23] and recently some activity in molecular recognition has been reported for these complexes [33,34].

The reaction of (+)-**9** with (UO_2_)(OAc)_2_ gave the uranyl complex (+)-**13** as a brown solid, whose structure was fully assigned from its ^1^H- and ^13^C-NMR spectra registered in (CD_3_)_2_SO. The lack of phenolic resonance in the protonic spectrum of **13** and the marked deshielding of the azomethinic and aminic protons (Δδ = +1.05 and Δδ = +1.47 ppm, respectively, compared with the free ligand **9**) were taken as an evidence of the nitrogen and oxygen coordination with the metal, which acts as an electron withdrawing group. In the ^13^C-NMR spectrum, the diagnostic resonances for the C-OH and C=N carbons were also shifted downfield upon complexation showing large Δδ values (Δδ = +9.13 and +5.53 ppm, respectively).

The ^1^H-NMR spectrum of **13** registered in CDCl_3_ showed broad resonances for all the protons, but two broad singlets in 1:1 ratio at 298 K were distinguishable for the azomethinic group and assigned to two diastereomeric conformers which slowly interconverted on the NMR time scale. By warming, the above resonances tended to collapse and at 338K a 80:20 ratio of the two conformers was measured. In the case of uranyl complex of **14**, with the same aminic moiety of (+)-**13** and taken as a reference, two conformers were also observed in CDCl_3,_ but not in (CD_3_)_2_SO, with a coalescence temperature of 300K. The slower interconversion rate of (+)-**13** compared with the (UO_2_)-**14 **complex could be ascribed to the presence of bulky ferrocenophane substituents.

The additional coordination of a molecule of ethanol to the uranium atom has been reported for some salen-uranyl complexes on the basis of elemental analyses data [35] and it can be easily detected by ^1^H-NMR but no evidence of such coordination was obtained for (+)-**13**. 

### 2.2. Circular dichroism spectra

Circular dichroism (CD) spectroscopy has been applied as a useful tool in the assignment of absolute configuration as well as conformational analysis of chiral molecules and biological polymers [36,37,38]. The development of quantum chemical CD calculations to be compared with experimental CD investigations has increased the reliability of the method and its extension to several classes of compounds [39,40]. The electronic absorption and the associated circular dichroism spectra have been reported for several salicylaldimine complexes with different cations and related with the coordination geometry of the central metal and the conformation of the diamine chelate ring [41,42,43,44].

The electronic and CD spectra of the free ligand (+)-**9** and the corresponding metal complexes were registered in CH_3_CN, except for (–)-**12** that was unsoluble in this solvent, and no substantial differences were observed in CH_3_Cl. 

In the UV-vis spectrum of (+)-**9** the azomethine π-π* transition band was observed at 338 nm and the benzene π-π* transitions gave rise to overlapping bands below 250 nm. Although two bands assigned to metal-ligand and intraligand charge transfer transitions could be expected for the ferrocene chromophore in the range 300-600 nm [45], they were not discernible, perhaps due to their low-intensity. 

**Figure 2:**
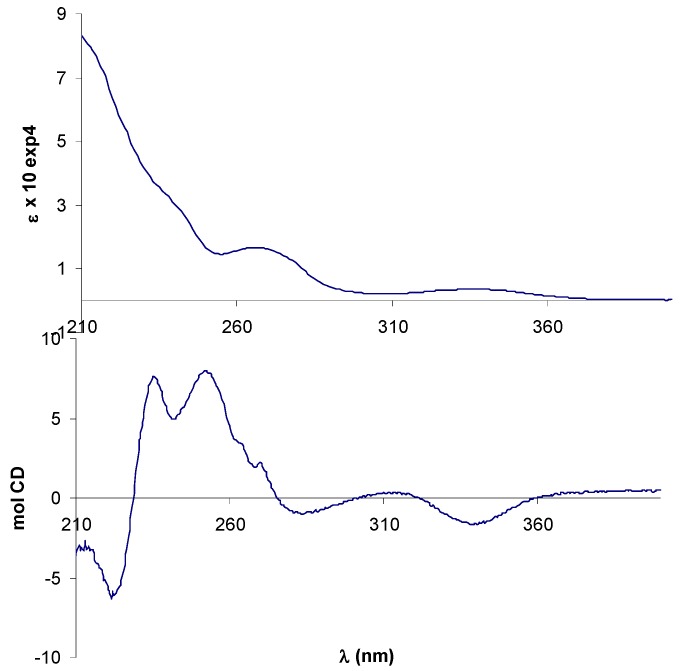
Electronic (up) and CD (bottom) spectra of free ligand (+)-**9** in CH_3_CN.

The CD spectrum of (+)-**9** displayed a negative Cotton effect (CE) associated with the π-π* transition of the azomethine chromophore, in agreement with the *R,R*-configuration of the diamine moiety [41]. The region 210-270 nm appeared better resolved in the CD than the UV-vis spectrum, but is clearly complicated by multiple CEs arising from benzenoid transitions involving salicylaldimine rings, phenyl rings on the (*R,R*)-dpen moiety and cyclopentadienyl rings (Figure 2).

**Figure 3:**
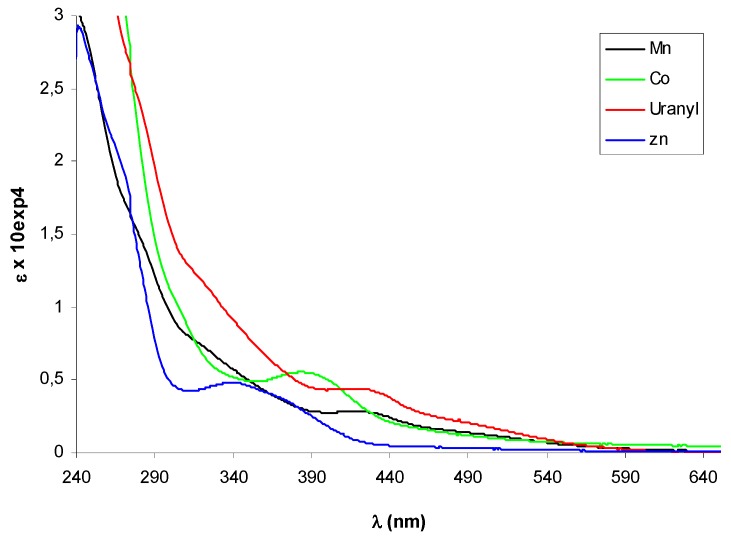
UV-vis spectra of metal complexes of (+)-**9**. The spectrum of Zn(II)-complex was registered in CHCl_3_, the others in CH_3_CN.

In the UV-vis spectra of complexes **10**-**13** additional absorption bands due to the presence of the metal could be expected in the 380-420 nm region, where ligand-metal charge transfer transitions (LMCT) as d-π∗ (azomethine) are usually observed, and in the 500-600 nm range, the typical absorption region for d-d transitions. The diagnostic azomethine π-π* transition was easily detected for the Zn-complex (–)-**12 ** appearing slight shifted with a maximum at 348 nm, but it seemed hidden by other bands in the spectra of the other complexes, that displayed a weaks band (ε < 6000) in the LMCT region (Figure 3).

**Figure 4:**
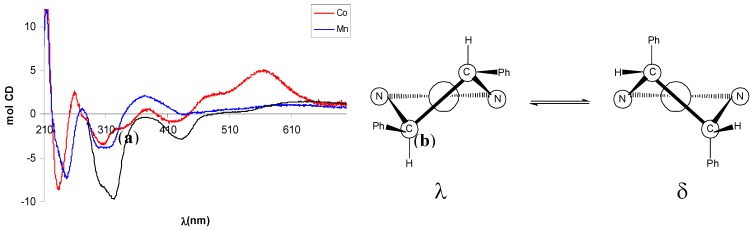
(a) CD spectra of Mn(III)-complex (–)-**10** and Co(II)-complex (+)-**11**. Black line refers to (–)-**10** in CHCl_3_. (b) Conformations and phenyl orientation for (*R,R*)-dpen diamine chelate ring.

In the CD spectrum of Mn(III)-complex (–)-**10** the negative CE around 320 nm, better resolved in CHCl_3_, can be related to a λ configuration of the five-membered ethylendiamine chelate ring on the basis of literature data [42]. Since the absolute configuration of the amine moiety is (*R*,*R*), a λ conformation of the chelate ring implies an equatorial disposition of the phenyl rings, in agreement with a repulsion between the axial phenyl groups and the apical acetate ligand that makes the other possible conformation δ less favoured (Figure 4).

The CD spectrum of Co(II)-complex (+)-**11** showed some degree of similarity with that of (–)-**10**, with a more intense CE related with d-d transitions at 560 nm (Figure 4). In analogy with tetradentate Schiff base Ni(II) or Cu(II) complexes [43,44], possessing the same square-planar geometry of Co(II) complexes, this band is diagnostic for the conformational assignment and a positive CE has been related with a shift of the conformational λ,δ-equilibrium toward the λ form. 

**Figure 5:**
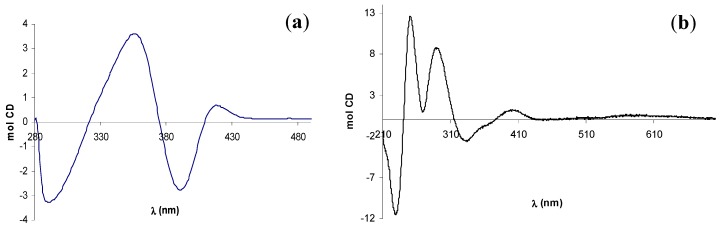
CD spectra of (a) zinc(II) complex (–)-**12** and (b) UO_2_-complex (+)-**13**.

The CD spectra of (–)-**12** and (+)-**13**, shown in Figure 5, are in good agreement with the reported data for the analogue complexes of **14 ** [35], but they not allow any conformational assignment since empirical rules establishing its relationship with the sign of a specific CE have not been developed for the salen complexes with these metals. 

## 3. Experimental

### 3.1. General methods

Reactions under microwave irradiation were performed in a 100 mL open vessel using a CEM Discover Benchmate equipped with temperature control device. Microprilled sodium hydroxide was purchased from Riedel-de-Haën. Diacetylferrocene and (1*R*,2*R*)-diphenylethylendiamine were available from Aldrich. Column chromatography was performed on Si 60 (230-400 mesh) silica gel using the specified eluants. ^1^H- and ^13^C-NMR spectra were registered in the specified deuterated solvents at 400 and 100 MHz, respectively. 2D-NMR were performed using standard Bruker programs. Chemical shifts (δ) are given as ppm relative to the residual solvent peak and coupling constants (*J*) are in Hz. In the NMR assignments, Cp and Ar refers to cyclopentadienyl and phenyl rings respectively. Melting points are uncorrected. Optical rotations were measured on a DIP 135 JASCO instrument. CD spectra were run at 20 °C on a JASCO J-810 spectropolarimeter using solutions in CH_3_CN ranging from 10^-4^ to 10^-5^ mol/L and 1 cm quartz cells. ESI mass spectra were obtained by employing an ES-MS Thermo-Finnigan LCQ-DECA spectrometer equipped with an ion trap analyzer and acquired in positive mode using 5V capillary voltage and 220 °C capillary temperature.

### 3.2. Synthesis of 1,5-dioxo-3-(p-methylbenzyloxyphenyl)[5]ferrocenophane (**4**)

To a solution of 4-hydroxybenzaldehyde in acetone (2.5 g, 20 mmol), 4-methylbenzylbromide (3.8 g, 20 mmol) and CsCO_3_ (9.1 g, 28 mmol) were added and the mixture left to react under stirring at rt. After 20 h the mixture was filtered and the solution was taken to dryness. The residue was purified on a Silica gel column and protected aldehyde **2** was recovered in 92% yield. In a 100 mL flask equipped with a reflux condenser, diacetylferrocene **1 **(2 mmol), aldehyde **2** and NaOH were mixed in 1:1:10 mol ratio and dissolved in 4:1 v/v EtOH/H_2_O (20 mL) and the mixture was subjected to 50W microwave irradiation for 30 min with 80 °C temperature setting [32]. After dilution with CH_2_Cl_2_ the mixture was extracted with satd. NH_4_Cl and the organic layer taken to dryness. The residue was washed twice with *n*-hexane/EtOAc to remove the reagents, leaving compound **4** as a gold yellow solid in 90% yield which was used without further purification. ^1^H-NMR (CDCl_3_) δ: 2.38 (s, 3H, CH_3_), 2.50 (m, 2H, CH_2_), 2.91 (t, 2H, *J* = 11.9, CH_2_), 4.31 (br t, 1H, CH), 4.58 (s, 2H, Cp), 4.62 (2H, Cp), 4.88 (s, 4H, Cp), 5.03 (s, 2H, CH_2_), 6.97 (d, 2H, *J* = 8.6, Ar), 7.21 (d, 2H, *J* = 7.9, Ar), 7.28 (d, 2H, *J* = 8.6, Ar), 7.34 (d, 2H, *J* = 7.9, Ar); Anal. Calcd. For C_29_H_26_FeO_3_: C, 72.81; H, 5.48. Found: C, 73.25; H, 5.54.

### 3.3. Synthesis of 4-([5]ferrocenophane-3-yl)-phenol (**6**)

To a solution of **4** (0.5 g, 1.1 mmol) in CH_2_Cl_2_ (150 mL) was added BH_3_/Me_2_S (2M in THF, 1.2 mL) and the reaction stirred overnight at room temperature. After quenching with MeOH (*caution*: gas evolution) the reaction mixture was extracted with sat. NH_4_Cl and the organic layer taken to dryness. The residue was dissolved in THF (20 mL) and to this solution Pd/C (50 mg) was added and the suspension stirred under H_2_ atmosphere (1.2 bar) at 60 °C for 3 h. As soon as quantitative conversion of the substrate was detected by TLC, the solid was removed by filtration through a short plug of Celite and the solution taken to dryness. The residue was purified by silica gel chromatography (*n*-hexane:EtOAc 8:2) to afford pure **6** as a gold-colored solid (0.3 g, 85% yield), mp 138-139 °C, ^1^H-NMR (CDCl_3_) δ: 1.93 (m, 2H, CH_2_), 2.02 (m, 2H, CH_2_), 2.21 (dt, 2H, *J* = 15.5, 5.1 and 5.1, CH_2_), 2.47 (ddd, 2H, *J* = 15.5, 10.1 and 3.9, CH_2_), 4.04 (s, 2H, Cp), 4.07 (br s, 5H, Cp and CH), 4.20 (s, 2H, Cp), 4.74 (s, 1H, OH), 6.82 (d, 2H, *J* = 8.3, Ar), 7.22 (d, 2H, *J* = 8.3, Ar); ^13^C-NMR (CDCl_3_) δ: 23.93 (CH_2_), 32.69 (CH_2_), 39.42 (CH), 66.20 (CpH), 67.16 (CpH), 68.03 (CpH), 69.24 (CpH), 89.33 (Cp), 115.20 (Ar-H), 128.73 (Ar), 140.57 (Ar), 153.65 (Ar-OH); ESI-MS: *m/z* 346.4 [M^+^]; Anal. Calcd. For C_21_H_22_FeO: C, 72.85; H, 6.40. Found: C, 73.58; H, 6.47.

### 3.4. Synthesis of 2-([5]ferrocenophane-3-yl)-phenol (**7**)

Starting from salicylaldehyde and applying the same reaction sequence described for compound **6, **the isomeric derivative **7** was obtained in 78% overall yield (referred to **1**).^ 1^H-NMR (CDCl_3_) δ: 2.00 (m, 4H, 2 x CH_2_), 2.28 (dt, 2H, *J* = 15.6, 4.9 and 4.9, CH_2_), 2.54 (ddd, 2H, *J* = 15.6, 10.3 and 4.6, CH_2_), 4.08 (s, 2H, Cp), 4.10 (s, 2H, Cp), 4.22 (s, 2H, Cp), 4.26 (s, 2H, Cp), 4.61 (m, 1H, CH), 4.88 (s, 1H, OH), 6.78 (d, 1H, *J* = 7.5, Ar), 6.95 (t, 1H, *J* = 7.5, Ar), 7.10 (t, 1H, *J* = 7.5, Ar), 7.31 (d, 1H, *J* = 7.5, Ar); ^13^C-NMR (CDCl_3_) δ: 24.08 (CH_2_), 31.71 (CH_2_), 32.00 (CH), 66.50 (CpH), 67.74 (CpH), 68.19 (CpH), 69.50 (CpH), 90.15 (Cp), 115.22 (Ar-H), 121.03 (Ar-H), 126.60 (Ar-H), 128.49 (Ar-H), 134.30 (Ar), 152.98 (Ar-OH); mp 134-135 °C, ESI-MS: *m/z* 346.6 [M^+^]; Anal. Calcd. For C_21_H_22_FeO: C, 72.85; H, 6.40. Found: C, 73.34; H, 6.46.

### 3.5. Synthesis of 4-([5]ferrocenophane-3-yl)-salicylaldehyde (**8**)

Compound **6** (300 mg, 0.77 mmol) was dissolved in CHCl_3_ (20 mL) and 13M KOH (2 mL) was added. The mixture was then refluxed for 24 h under vigorous stirring and the reaction monitored by TLC analysis (*n*-hexane:EtOAc 85:15). The reaction was then quenched at about 25% conversion of substrate, when **8** was detected as the sole product, by addition of H_2_O and CH_3_COOH to acidic pH. After extraction the organic layer was dried over MgSO_4_ and taken to dryness. Column chromatography on Si gel (*n*-hexane:EtOAc 85:15) gave pure **8** (60 mg, 21% yield) and the unreacted **6 **(210 mg, 70% yield), which was then recycled. mp 139-140 °C; ^1^H-NMR (CDCl_3_) δ: 1.96 (m, 2H, CH_2_), 2.06 (m, 2H, CH_2_), 2.22 (ddd, 2H, *J* = 15.6, 6.2 and 4.3, CH_2_), 2.48 (ddd, 2H, *J* = 15.6, 10.1 and 6.4, CH_2_), 4.04 (s, 2H, Cp), 4.09 (s, 4H, Cp), 4.21 (br s, 3H, Cp and CH), 6.99 (d, 1H, *J* = 8.5, Ar), 7.49 (d, 1H, *J* = 2.2, Ar), 7.53 (dd, 1H, *J* = 8.5 and 2.2), 9.95 (s, 1H, CHO), 10.90 (s, 1H, OH); ^13^C-NMR (CDCl_3_) δ: 23.82 (CH_2_), 32.45 (CH_2_), 39.36 (CH), 66.21 (CpH), 67.11 (CpH), 68.11 (CpH), 69.06 (CpH), 89.99 (Cp), 117.55 (Ar-H), 120.50 (Ar), 131.97 (Ar-H), 136.31 (Ar-H), 139.73 (Ar), 160.25 (Ar-OH) 196.61 (CHO); ESI-MS: *m/z* 374.3 [M^+^]; Anal. Calcd. For C_22_H_22_FeO_2_: C, 70.60; H, 5.93. Found: C, 70.98; H, 6.01.

### 3.6. Synthesis of (+)-(R,R)-N,N’-bis[4-([5]ferrocenophane-3-yl)-salicylidene]-1,2-diphenylethyle-nediamine [(+)-9]

To a solution of aldehyde **8 **(47 mg, 0.126 mmol) in absolute ethanol (100 mL) (1*R*,2*R*)-1,2-diphenylethylenediamine (13.3 mg, 0.063 mmol) was added and the reaction mixture maintained under stirring at room temperature for 2 days. When the TLC analysis indicated the disappearance of the aldehyde, the solvent was removed *in vacuo* and the residue applied to a silica gel column (*n*-hexane:EtOAc 9:1) to give pure (+)-**9** (55 mg, 0.059 mmol, 94% yield) as a pale orange solid, mp 139-140 °C; [α]_D_ = +20.2 (*c* 0.48, CHCl_3_); ^1^H-NMR (CDCl_3_) δ: 1.85 (m, 4H, -CH_2_), 1.96 (m, 4H, -CH_2_), 2.16 (m, 4H, -CH_2_), 2.41 (m, 4H, -CH_2_), 3.98 (br s, 2H, Cp), 4.00 (br s, 2H, Cp), 4.07 ( s, 10H, Cp and CH), 4.20 (m, 4H, Cp), 4.76 (s, 2H, CH-N), 6.95 (d, 2H, *J* = 8.3, Ar), 7.11 (m, 2H, Ar), 7.24 (m, 12H, Ar), 8.41 (s, 2H, CH=N), 13.15 (s, 2H, OH); ^13^C-NMR (CDCl_3_) δ: 23.88 (CH_2_), 32.50 (CH_2_), 32.56 (CH_2_), 39.35 (CH), 66.20 (CpH), 67.16 (CpH), 68.00 (CpH), 69.20 (CpH), 69.25 (CpH), 80.39 (CH-N), 89.20 (Cp), 116.77 (Ar-H), 118.40 (Ar), 127.58 (Ar-H), 127.94 (Ar-H), 128.36 (Ar-H), 130.64 (Ar-H), 132.00 (Ar-H), 138.45 (Ar), 139.48 (Ar), 159.08 (Ar-OH), 166.46 (C=N); ESI-MS: *m/z* 924.2 [M^+^], 462.4 [M^++^]; Anal. Calcd. For C_58_H_56_Fe_2_N_2_O_2_: C, 75.33; H, 6.10; N, 3.03. Found: C, 75.91; H, 6.16; N, 3.08.

### 3.7. General procedure for the synthesis of metal complexes

To a solution of (+)-**9** (50 mg, 54 mmol) in absolute EtOH (10 mL), the appropriate M(OAc)*_n_*·*x*H_2_O (54 mmol, M = Mn(III), Co(II) or [UO_2_]^2+^) was added and the mixture left to react at rt under stirring until TLC analysis showed the complete conversion of substrate. The complex was separated from the solution by filtration and air-dried (80–90% yield)

*Mn(III)-complex* (–)-**10**: [α]_D_ = –642.1 (*c* 0.05, CHCl_3_); ESI-MS: *m/z* 1023.3 [M + EtOH]^+^; CD: λ (Δε) 625 (+1.08), 370 (+2.08), 306 (–3.82), 268 (+0.38), 243 (–7.16); Anal. Calcd. for C_60_H_57_Fe_2_MnN_2_O_4_: C, 69.51; H, 5.54; N, 2.70. Found: C, 69.48; H, 5.50; N, 2.67.

*Co(II)-complex* (+)-**11**: [α]_D_ = +610.2 (*c* 0.05, CHCl_3_); ESI-MS: *m/z* 981.2 [M]^ +^, 490.8 [M]^ ++^; CD: λ (Δε) 562 (+4.90), 470 (+1.83), 416 (–0.77), 372 (+0.42), 300 (–3.78), 257 (+2.07), 232 ( –8.31); Anal. Calcd. For C_58_H_54_CoFe_2_N_2_O_2_ : C, 70.69; H, 5.54; N, 2.85. Found: C, 70.66; H, 5.49; N, 2.82.

*UO_2_ complex* (+)-**13**: [α]_D_ = +142.7 (*c* 0.15, C_6_H_6_); ^1^H-NMR (*d_6_*-DMSO) δ: 1.91 (m, 4H, -CH_2_), 2.03 (m, 4H, -CH_2_), 2.12 (m, 4H, -CH_2_), 2.42 (m, 4H, -CH_2_), 4.07 (br s, 10H, Cp and -CH), 4.10 (s, 4H, Cp), 4.19 (s, 4H, Cp), 6.22 (s, 2H, CH-N), 6.97 (d, 2H, *J* = 8.6, Ar), 7.20 (m, 6H, Ar), 7.49 (s, 2H, Ar), 7.62 (dd, 2H, *J* = 2.0 and 8.6, Ar), 7.66 (d, 4H, *J* = 7.5, Ar), 9.44 (s, 2H, CH=N); ^13^C-NMR (*d_6_*-DMSO) δ: 23.94 (CH_2_), 32.98 (CH_2_), 38.81 (CH), 66.35 (CpH), 67.34 (CpH), 68.17 (CpH), 69.22 (CpH), 80.61 (CH-N), 89.00 (Cp), 120.70 (Ar-H), 123.22 (Ar), 127.60 (Ar-H), 127.79 (Ar-H), 128.68 (Ar-H), 133.84 (Ar-H), 135.08 (Ar-H), 136.06 (Ar), 142.2 (Ar), 168.21 (Ar-O), 171.99 (C=N); ESI-MS: *m/z* 1192.3 [M]^+^; CD: λ (Δε) 575 (+0.68), 398 (+1.16), 331 (–2.52), 287 (+8.60), 250 (+12.45), 228 (–11.48); Anal. Calcd. For C_58_H_54_Fe_2_N_2_O_4_U: C, 58.40; H, 4.56; N, 2.35. Found: C, 58.38; H, 4.49; N, 2.31.

### 3.8. Synthesis of Zn(II) complex (-)-**12**

A 2M solution of Et_2_Zn in toluene (0.030 mL, 60 mmol) was added to a solution of (+)-**9** (50 mg, 54 mmol) in toluene (5 mL) and the mixture stirred at rt overnight. The solvent was removed under vacuum, the residue suspended in CHCl_3_ and the solid removed by filtration. The final solution was taken to dryness to give pure complex in quantitative yield: [α]_D_ = – 21.3 (*c* 0.3, CHCl_3_); ^1^H-NMR (*d_6_*-DMSO) δ: 1.77 (m, 4H, -CH_2_), 1.92 (m, 4H, -CH_2_), 2.05 (m, 4H, -CH_2_), 2.37 (m, 4H, -CH_2_), 3.86 (m, 2H, CH), 4.00 (s, 4H, Cp), 4.03 (s, 4H, Cp), 4.06 (s, 4H, Cp), 4.14 (s, 4H, Cp), 5.05 (s, 2H, CH-N), 6.64 (d, 2H, *J* = 8.7, Ar), 7.00 (d, 2H, *J* = 2.2, Ar), 7.17 (dd, 2H, *J* = 8.7 and 2.2, Ar), 7.26 (br t, 2H, Ar), 7.36 (t, 4H, *J* = 7.5, Ar), 7.44 (d, 4H, *J* = 7.5, Ar), 8.36 (s, 2H, CH=N); ^13^C-NMR (*d_6_*-DMSO) δ: 23.87 (CH_2_), 32.56 (CH_2_), 39.23 (CH), 66.20 (CpH), 67.22 (CpH), 68.02 (CpH), 69.33 (CpH), 73.13 (CH-N), 89.40 (Cp), 118.9 (Ar), 123.09 (Ar-H), 127.85 (Ar-H), 127.92 (Ar-H), 128.87 (Ar-H), 131.4 (Ar), 133.44 (Ar-H), 133.56 (Ar-H), 142.37 (Ar), 170.32 (Ar-O), 170.65 (C=N); ESI-MS: *m/z* 987.3 [M]^+^, CD: λ (Δε) 416 (+0.63), 389 (–2.74), 353 (3.58), 289 (–3.25); Anal. Calcd. For C_58_H_54_Fe_2_N_2_O_2_Zn : C, 70.50; H, 5.51; N, 2.83. Found: C, 70.46; H, 5.48; N, 2.80.

## 4. Conclusion

A novel salen ligand (+)-**9** bearing two [5]ferrocenophane *para*-substituents on the salicylideneamine rings has been synthesized starting from readily available diacetylferrocene, *p*-hydroxybenzaldehyde and (*R*,*R*)-dpen and its complexes with four different metals have been also prepared and characterised. The evaluation of the use of these complexes as ligands in asymmetric synthesis is currently under investigation.
